# Long non-coding RNA NEAT1 facilitates the growth, migration, and invasion of ovarian cancer cells via the let-7 g/MEST/ATGL axis

**DOI:** 10.1186/s12935-021-02018-3

**Published:** 2021-08-20

**Authors:** Lili Yin, Yu Wang

**Affiliations:** Department of Obstetrics and Gynecology, Shengjing Hospital of China Medical University, No. 36, Sanhao Street, Heping District, Shenyang, Liaoning Province 110004 P.R. China

**Keywords:** Ovarian cancer, Long non-coding RNA nuclear-enriched abundant transcript 1, microRNA let-7 g, Mesoderm specific transcript, Adipose triglyceride lipase, Migration, Apoptosis

## Abstract

**Background/Aim:**

Growing evidence indicates a significant role of long non-coding RNA (lncRNA) nuclear-enriched abundant transcript 1 (NEAT1) in ovarian cancer, a frequently occurring malignant tumor in women; however, the possible effects of an interplay of NEAT1 with microRNA (miRNA or miR) let-7 g in ovarian cancer are not known. The current study aimed to investigate the role of the NEAT1/let-7 g axis in the growth, migration, and invasion of ovarian cancer cells and explore underlying mechanisms.

**Methods:**

NEAT1 expression levels were examined in clinical tissue samples and cell lines. The relationships between NEAT1, let-7 g, and MEST were then analyzed. Gain- or loss-of-function approaches were used to manipulate NEAT1 and let-7 g. The effects of NEAT1 on cell proliferation, migration, invasion, and apoptosis were evaluated. Mouse xenograft models of ovarian cancer cells were established to verify the function of NEAT1 in vivo.

**Results:**

NEAT1 expression was elevated while let-7 g was decreased in ovarian cancer clinical tissue samples and cell lines. A negative correlation existed between NEAT1 and let-7 g, whereby NEAT1 competitively bound to let-7 g and consequently down-regulate let-7 g expression. By this mechanism, the growth, migration, and invasion of ovarian cancer cells were stimulated. In addition, let-7 g targeted mesoderm specific transcript (MEST) and inhibited its expression, leading to promotion of adipose triglyceride lipase (ATGL) expression and inhibition of ovarian cancer cell growth, migration, and invasion. However, the effect of let-7 g was abolished by overexpression of MEST. Furthermore, silencing of NEAT1 decreased the xenograft tumor growth by decreasing MEST while up-regulating let-7 g and ATGL.

**Conclusions:**

Cumulatively, the findings demonstrated that NEAT1 could promote malignant phenotypes of ovarian cancer cells by regulating the let-7 g/MEST/ATGL signaling axis. Therefore, NEAT1 can be regarded as an important molecular target and biomarker for ovarian cancer.

**Supplementary Information:**

The online version contains supplementary material available at 10.1186/s12935-021-02018-3.

## Background

Ovarian cancer is one of the leading causes of cancer death among women and the most lethal gynecologic malignancy [1]. Unfortunately, ovarian cancer has no specific clinical manifestations in the early stage and most patients present metastatic and invasive cancer cells at the time of diagnosis [2]. The established risk factors for this disease include oral contraceptive use, height and body mass index, dietary factors, smoking, and insufficient physical activity [3]. The foremost treatment for ovarian cancer is debulking surgery followed by cisplatin chemotherapy [4]. Concerningly, the 5-year survival rate of advanced ovarian cancer less than 20% [5] on account of frequent relapses, late diagnosis, development of drug resistance and side effects of chemotherapy [6]. It is thus imperative to extensively explore the mechanisms underlying ovarian cancer, in order to identify most suitable targets for ovarian cancer treatment.

Long non-coding RNAs (lncRNAs) are a type of noncoding RNA that comprise of longer than 200 nucleotides sequences, and hold strong promise as biomarkers and therapeutic targets for cancer due to their genome-wide expression in various tissues and the tissue specific expression characteristics [7]. LncRNA nuclear paraspeckle assembly transcript 1 (NEAT1) has been established as a structural component of paraspeckles, which modulates multiple genes’ expression through nuclear retention [8]. In a previously conducted study, NEAT1 was confirmed to accelerate the metastasis of ovarian cancer cells [9]. Another study has also reported the implication of NEAT1 in the tumorigenesis and development of ovarian cancer [10]. Additionally, NEAT1 knockdown has been reported to suppress ovarian cancer cell proliferation, colony formation, migration, and invasion while stimulating cell apoptosis [11]. Furthermore, NEAT1 can target microRNA let-7 g-5p (miR let-7 g-5p) and negatively regulate its expression in glioblastoma [12]. Another class of molecular regulators, miRNAs are a class of small single-stranded non-coding RNAs and exert crucial roles in the initiation and progression of cancer, acting either as oncomiRs or as tumor suppressors through several molecular mechanisms [13]. In particular, let-7 g has been involved in the development of breast cancer and hepatocellular carcinoma [14, 15]. Additionally, let-7 g functions as a predictive biomarker for chemoresistance and tumor inhibitor in human epithelial ovarian cancer [16]. Furthermore, poor expression of let-7 g has been extensively associated with the resistance of epithelial ovarian cancer cells to chemotherapy [16]. The starBase database (starbase.sysu.edu.cn/) employed in the current study revealed the presence of binding sites between let-7 g and mesoderm specific transcript (MEST). MEST is a cancer-related maternal imprinted gene, which is essential for normal early embryonic progression [17]. Additionally, an earlier study has revealed that MEST, whose promoter is markedly hypomethylated in resistant relative to sensitive xenografts, participates in ovarian cancer [18]. During human adipocyte differentiation, silencing of MEST is shown capable of elevating the expression of adipose triglyceride lipase (ATGL) [19], which is the major enzyme responsible for the initial step of triglyceride degradation and an important enzyme in maintaining whole-body energy homeostasis [20]. Moreover, decreased mRNA expression of ATGL has shown correlation with short survival times in patients with ovarian cancer [21]. Based on the aforementioned findings, we speculated NEAT1, let-7 g, MEST and ATGL may interact and such interaction could be important in ovarian cancer pathology. To address this hypothesis, the current study aimed to explore their correlation and the mechanisms mediating the effect of the NEAT1/let-7 g/MEST/ATGL axis on the growth, migration, and invasion of ovarian cancer.

## Materials and methods

### Ethics statement

The current study protocols were approved by the Ethics Committee of Shengjing Hospital of China Medical University (approval number: 2021PS272K) and conducted in strict accordance with the *Declaration of Helsinki*. Signed informed consent was obtained from all participants before sample collection. All animal experiments were conducted with the approval of the Ethics Committee of Shengjing Hospital of China Medical University (approval number: 2021PS330K) and in accordance to the Guide for the Care and Use of Laboratory Animal published by the US National Institutes of Health. Extensive efforts were made to ensure minimal suffering of the animals included in the study.

### Microarray analyses of ovarian cancer

The expression dataset of ovarian cancer GSE46169 was obtained from the Gene Expression Omnibus (GEO) database (https://www.ncbi.nlm.nih.gov/geo/), including 3 normal samples and 27 tumor samples. With the normal samples used as controls, the R language "limma" package was applied for differential analysis. The False Discovery Rate (FDR) method was used for *p* value correction, with |logfold change|> 2 and FDR < 0.05 regarded as the screening criteria for differentially-expressed genes. Then, the target genes of the selected miRNA were predicted by starBase database (starbase.sysu.edu.cn/) and mirwalk website (http://mirwalk.umm.uni-heidelberg.de/). Next, we downloaded the list of up-regulated genes in ovarian cancer from the Cancer Genome Atlas (TCGA)database through the GEPIA website (http://gepia.cancer-pku.cn/), followed by intersection of these target genes and up-regulated genes to obtain the possible regulatory mRNA. Subsequently, the starBase database (http://starbase.sysu.edu.cn/panCancer.php) was performed to verify the correlation between the candidate target genes and miRNAs to finally determine the target gene selected in this study. Then the correlation between the expressions of MEST and ATGL (patatin like phospholipase domain containing 2) in TCGA ovarian cancer was analyzed.

### Study subjects

In the duration from November 2017 to May 2018, ovarian cancer tissues and adjacent normal tissues from 68 ovarian cancer patients (21–64 years) treated at the department of obstetrics and gynecology of Shengjing Hospital of China Medical University were collected. The diagnosis of these pathological specimens was confirmed at the department of pathology, and relevant clinical data were collected through a retrospective review of patient files. The clinical characteristics of the enrolled patients are listed in Additional file 1: Table S1. The patients with non-epithelial neoplasms, preoperative treatment, and those not treated surgically were excluded from the study. Additionally, borderline ovarian tumors were also excluded from this study. The obtained tissue sections were frozen in liquid nitrogen and stored at − 80℃ [22].

### Cell culture, grouping and transfection

Normal ovarian cancer epithelial cell line IOSE80 and ovarian cancer cell lines of ES2, A2780, HO8910, and SKOV3 were purchased from American Type Culture Collection (Manassas, VA, USA). All cell lines were subjected to cell line authentication by short tandem repeat (STR) profiling (Additional file 1: Table S2). The cells were cultured in Dulbecco’s modified Eagle’s medium (DMEM; Gibco, Carlsbad, CA, USA) containing 10% fetal bovine serum (FBS), 50 U/mL penicillin and 50 g/mL streptomycin (Gibco). All cell lines were cultured in a humid environment at 37℃ with 5% CO_2_. According to the known sequences of NEAT1, let-7 g, MEST, and ATGL in National Center for Biotechnology Information (NCBI), the plasmids needed for the experiment were constructed (Shanghai Sangon Biotechnology Co., Ltd., Shanghai, China). Subsequently, the small interfering RNA targeting NEAT (si-NEAT) was used to down-regulate the expression of NEAT, with si-negative control (NC) serving as the control. let-7 g mimic was used to overexpress let-7 g expression, while let-7 g inhibitor was used to down-regulate let-7 g expression, with NC mimic and NC inhibitor serving as the controls, respectively. Additionally, the oe-MEST was applied to overexpress MEST and oe-NC was regarded as the control. The cells at third passage were detached using trypsin, and subsequently seeded into a 24-well plate (initial concentration was 2 × 10^6^ cells/well) and cultured into a monolayer. Consequently, the cells in the logarithmic phase were divided into 2 groups upon reaching 75% confluence. Cell transfection was performed in accordance with standard instructions using Lipofectamine 2000 reagents (Invitrogen, Carlsbad, CA, USA) after the medium was discarded.

### Reverse transcription quantitative polymerase chain reaction (RT-qPCR)

At 24 h post transfection, the total RNA from the tissues was extracted using TRIzol reagents (No.16096020, Thermo Fisher Scientific Inc., New York, USA). Next, 5 µg of the extracted total RNA was reverse transcribed into complementary DNA (cDNA) using a TaqMan™ MicroRNA Reverse Transcription Kit (Thermo Fisher Scientific Inc.) according to kit instructions. With the cDNA as the template, the amplification of let-7 g was carried out using the TaqMan MicroRNA Assay and the TaqMan® Universal PCR Master Mix, and the amplification of NEAT1, MEST and ATGL was conducted using the TaqMan Gene Expression Assays protocol (Applied Biosystems, Foster City, CA, USA). U6 and glyceraldehyde-3-phosphate dehydrogenase (GAPDH) were utilized as internal reference genes to normalize the results. Three parallel triplicate wells were used for each biological replicate and RT-qPCR was performed. The primers are shown in Additional file 1: Table S3. The differences in expression levels of the target genes between the experimental group and the control group were calculated using the 2^−ΔΔCt^ method.

### Western blot analysis

Total protein was extracted from the cells and tissues using the radio-immunoprecipitation assay (RIPA) lysis buffer (R0010, Beijing Solarbio Science & Technology Co., Ltd., Beijing, China). Protein concentration was determined using the bicinchoninic acid protein assay kit (GBCBIO Technologies Inc., Guangzhou, China). Subsequently, 40 μg of protein was separated by 10% sodium dodecyl sulfate polyacrylamide gel electrophoresis, and transferred onto polyvinylidene difluoride membranes (Millipore, Billerica, MA, USA). The membranes were then blocked with Tris-buffered saline with Tween 20 solution containing 5% bovine serum albumin, and incubated with diluted primary rabbit antibodies against MEST (1: 1000, ab230114), ATGL (1: 1000, ab207799) and GAPDH (ab9485, 1: 2500, Abcam Inc., Cambridge, UK) at 4℃. The following day, the membranes were incubated with secondary antibody goat anti-rabbit immunoglobulin G (IgG) (ab150077, 1: 1000, Abcam). Next, enhanced chemiluminescence reagents were used for membrane development. The protein bands were exposed and developed on the Image Quant LAS 4000C gel imager (GE, Boston, USA).

### Immunohistochemistry

The paraffin sections of transplanted tumor were baked for 30 min at 60 °C in an oven, dewaxed, hydrated, immersed in xylene I and II and gradient alcohol for 5 min, and finally rinsed under tap water for 2 min. Subsequently, microwave antigen retrieval was conducted with 1 mM Tris-ethylene diamine tetraacetic acid (pH 8.0). Following cooling to room temperature, the sections were then treated with 3% H_2_O_2_-methanol for 10 min to block endogenous peroxidase (POD) activity. The sections were subsequently immunostained with primary antibody against MEST (1: 100, ab230114) and ATGL (1: 500, ab207799, Abcam) overnight at 4 °C. Following three washes with 0.1% phosphate buffer solution Tween-20 (5 min/time), on the following day, the sections were further immunostained with polymer enhancer (PV-9000, ZSGB-Bio, Beijing, China) for 20 min and then with enzyme-labeled anti-rat/rabbit polymers (PV-9000, ZSGB-Bio) for 30 min. Subsequently, the sections were developed for 5 min with 3,3'-diaminobenzidine tetrahydrochloride (DAB), which was terminated by the addition of distilled water. The sections were then counterstained by hematoxylin, differentiated, blued, dehydrated, cleared and sealed with neutral gum. Finally, the sections were observed and photographed under an inverted microscope (CX41, Olympus Optical Co., Ltd., Tokyo, Japan).**

### Dual luciferase reporter gene assay

The binding sites of NEAT1 and let-7 g as well as MEST and let-7 g were predicted using the starBase database and a dual luciferase reporter gene assay was employed to verify the target relationships between let-7 g and NEAT1 as well and that between MEST and let-7 g. According to the binding sequence of let-7 g and NEAT1 as well as the 3′ untranslated region (UTR) of MEST mRNA and let-7 g, the target sequence and mutation sequence were each designed. The target sequence was subsequently introduced into the pMIR-reporter (Beijing HuaYueyang Biotechnology Co. Ltd., Beijing, China) by endonuclease sites SpeIand Hind III. The mutation sequence was implemented with endonuclease cleavage by restriction enzyme and the target fragment was introduced into pMIR-reporter plasmid by T4 DNA ligase. The properly sequenced luciferase reporter plasmids wild type (WT) and mutant type (MUT) were then co-transfected with let-7 g mimic and NC-mimic into HEK293T cells (Shanghai Beinuo Biotechnology Co., Ltd., Shanghai, Beijing). After 48 h of transfection, the cells were harvested and lysed. Next, the luciferase activity was detected using a Glomax20/20 luminometer (Promega, Madison, WI, USA) of the dual luciferase detection system (Promega). The relative luciferase activity was calculated using the formula: firefly luciferase activity/renilla luciferase activity.

### RNA binding protein immunoprecipitation (RIP) assay

The binding of NEAT1 and Argonaute2 (AGO2) protein was tested using the RIP kit (Millipore). Ovarian cancer cells were cleaned with pre-cooled phosphate buffered saline (PBS) and the supernatant was removed and lysed using an equal volume of RIPA cell lysis buffer (P0013B, Beyotime Institute of Biotechnology, Shanghai, China) for 5 min, and further centrifuged for 10 min at 14,000 rpm (4 °C) to obtain the supernatant. A part of the cell extraction liquid was used as input, and another part was incubated with antibody for further coprecipitation. The magnetic bead-antibody compound was suspended with 900 μL RIP wash buffer and incubated with 100 μL cell extraction liquid at 4 °C. The sample and input were then detached using protease K to extract RNA for further PCR detection. The antibody used by RIP was rabbit poly-anti AGO2 (ab32381, 1: 10,000, Abcam) (30 min). Rabbit poly-anti IgG (ab202985, 1: 1000, Abcam) was used as the NC.

### RNA-pull down assay

Ovarian cancer cells were transfected with biotinylated WT-NEAT1 and biotinylated MUT-NEAT1 (each 50 nM) for 2 d, and then gathered and cleaned using PBS. The cells were then incubated for 10 min with special cell lysate. The residual lysate was incubated with M-280 streptavidin magnetic beads (Sigma, St. Louis, MO, USA) and pre-coated with RNase-free and yeast tRNA (Sigma) for 3 h, then cleaned twice with cool lysate, three times with low salt buffer and once with high salt buffer. The antagonistic let-7 g probe was set as the NC. The total RNA was extracted and the expression of let-7 g was determined using RT-qPCR.

### Flow cytometry

After 48 h of transfection, the cell medium was transferred to a 15 mL tapered tube and placed on ice. Cells in the culture plate were washed using 2 mL PBS, incubated with 0.5 mL 0.25% trypsin and observed under a microscope. Cells were then detached from the culture plate wall, and suspended in the medium at a density of 1 × 10^6^ cells/mL. The cell suspension (0.5 mL, 1 × 10^6^ cells/mL) was then transferred to a clean centrifuge tube, which was added with staining solution, and suspended in 0.5 mL pre-cooled 1 × binding buffer. The sample was then incubated for 15 min with 5 μL Annexin V-fluorescein isothiocyanate and 10 μL propidium iodide. Finally, the results were analyzed using a flow cytometer (FACSVerse/Calibur/AriaIISORP, BD, USA). All the above-mentioned kits were purchased from Beyotime Biotechnology.

### Transwell assay

Migration test: ovarian cancer cells in the logarithmic phase were starved for 24 h, and on the following day, they were detached, centrifuged, and suspended to a final concentration of 2 × 10^5^ cells/mL. Subsequently, 0.2 mL of the cell suspension was added to the upper Transwell chamber, and 700 μL pre-cooled DMEM containing 10% FBS was added to the lower chamber, followed by culture in a 37 °C incubator. The Transwell chamber was then removed after 1 d, and the cells in the upper chamber and basement membrane were wiped off using wet cotton swabs. The cells were fixed for 30 min with methanol, stained with 0.1% crystal violet staining solution for 20 min, air-dried, and then observed and photographed under an inverted microscope in five randomly selected fields of view to count the number of permeating cells.

Invasion test: the Matrigel was allowed to stand at 4 °C overnight, and on the following day, the cells were diluted using serum-free medium at 1: 9 to a final concentration of 1 mg/mL. Matrigel (40 μL) was added on the polycarbonate membrane of each 24-well Transwell upper chamber, then added with 70 μL/chamber pure DMEM and incubated for 30 min to enable hydration of the Matrigel. Subsequently, the cells were starved for 1 d, detached, centrifuged, and suspended in DMEM without FBS to a final concentration of 2.5 × 10^5^ cells/mL. The cell suspension (0.2 mL) was added to the upper chamber whose basement membrane was hydrated. The lower chamber was added with 700 μL pre-cooled DMEM containing 10% FBS, and then cultured for 24 h in a saturated humidity incubator. Thereafter, the Transwell chamber was removed, and the cells in the chamber and basement membrane were wiped off using wet cotton swabs. Following this, the cells were fixed for 30 min with methanol, stained with 0.1% crystal violet staining solution for 20 min, air-dried, and then observed and photographed under an inverted microscope in five randomly selected fields of view to count the number of permeating cells.

### Tumor xenograft in nude mice

Thirty-two female BALA/c nude mice (aged 4–6 weeks, weighing 18–20 g) were obtained from Hunan SJA Laboratory Animal Co., Ltd. (Hunan, China) (http://www.hnsja.com/). The mice were placed in a specific pathogen-free grade environment. Subsequently, the mice were divided into 4 groups (8 mice in each group): short hairpin RNA (sh)-NC + NC-inhibitor group (5 × 10^3^ SKOV3 cells/mouse), sh-NEAT1 + NC-inhibitor group (5 × 10^3^ SKOV3 cells/mouse), sh-NC + let-7 g-inhibitor group (5 × 10^3^ SKOV3 cells/mouse), and sh-NEAT1 + let-7 g-inhibitor group (5 × 10^3^ SKOV3 cells/mouse). Injections were started at the 5^th^ d and finished at the 40^th^ d. The tumor volume was measured every 5 days. The mice were euthanized by carbon dioxide asphyxiation after the experiment. The volume of transplanted tumor was calculated using the formula: V = (A × B^2^)/2 (A indicates length and B indicates width, mm^3^), with the curve of the average volume at each time point plotted.

### Terminal deoxynucleotidyl transferase-mediated deoxyuridine triphosphate-biotin nick end-labeling (TUNEL) staining

The tumor tissues were fixed in 4% paraformaldehyde, paraffin-embedded, and then cut into 5-mm-thick slices. Five tissue slices were dewaxed, hydrated, dropped with 1% protease K diluent (50 μL), and incubated in a 37℃ incubator for 30 min. Subsequently, the slices were added with 0.3% H_2_O_2_ methanol to eliminate endogenous POD activity and further incubated for 1 h. Next, TUNEL reaction solution was added and incubated in a wet box at 37℃ for 1 h in the dark, followed by addition of 50 μL Converter-POD for 30 min of incubation in a wet box at 37℃ for 1 h in the dark. Thereafter, the slices were developed with 2% DAB for 15 min and observed under a light microscope. When the nuclei were seen as brownish yellow, the reaction was terminated by the addition of distilled water. The slices were then counterstained using hematoxylin, dehydrated by gradient alcohol (50%, 70%, 90% and 100%), cleared by xylene, and sealed by neutral gum. The slices were then observed under an optical microscope in ten randomly selected fields of view from each slice. Cells with brownish-yellow nuclei were apoptotic positive cells and cells with blue nuclei were normal cells.

### Statistical analysis

All data were processed using SPSS 21.0 software (IBM Corp., Armonk, NY, USA). Measurement data were expressed as mean ± standard deviation. Comparisons between cancer tissues and adjacent normal tissues were performed using paired *t*-test, and other two-group comparisons were conducted by independent sample *t*-test. Data comparisons between multiple groups were conducted using one-way analysis of variance (ANOVA) and Tukey's post hoc test. Data from multiple groups at different time points were compared using repeated measures ANOVA and Bonferroni post hoc test. Pearson correlation analysis was used for correlation analysis between indicators in clinical samples. *p* < 0.05 was considered statistically significant.

### Results

### NEAT1 is up-regulated while let-7 g is decreased in ovarian cancer

**Fig. 1:**
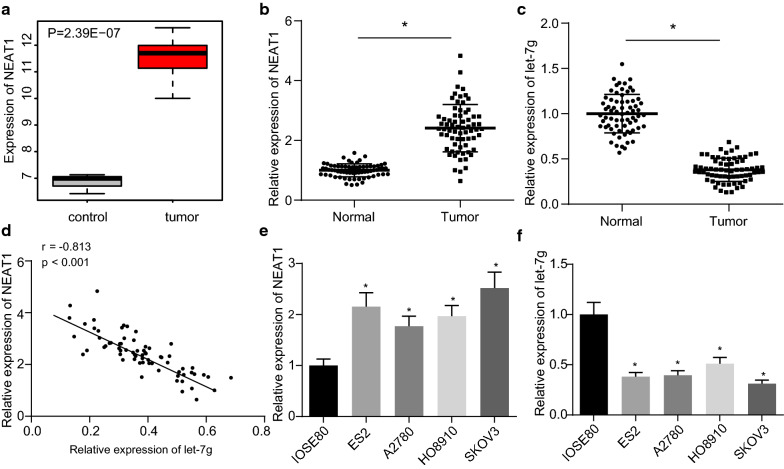
NEAT1 expression is increased and let-7 g is decreased in ovarian cancer. **A** Differential expression of NEAT1 in normal samples and ovarian cancer samples in the GSE46169 dataset retrieved from the GEO database. **B** Expression of NEAT1 in ovarian cancer tissues and adjacent normal tissues determined by RT-qPCR, n = 68. **C** Expression of let-7 g in ovarian cancer tissues and adjacent normal tissues determined by RT-qPCR, n = 68. **D** Pearson correlation analysis of NEAT1 expression level and let-7 g expression level in clinical samples, n = 68. **E** Expression of NEAT1 in normal ovarian epithelial cell line IOSE80 and ovarian cancer cell lines ES2, A2780, SKOV3 and HO8910 determined by RT-qPCR. F, Expression of let-7 g in normal ovarian epithelial cell line IOSE80 and ovarian cancer cell lines ES2, A2780, SKOV3 and HO8910 determined by RT-qPCR. In figure **A**, **B** * *p* < 0.05 vs. adjacent normal tissues. In figure **D**, **E**, * *p* < 0.05 vs. IOSE80 cell line. The measurement data were expressed as mean ± standard deviation. Comparisons between two groups were conducted using paired *t*-test, while comparison between multiple groups were performed by one-way ANOVA followed by Tukey's post hoc test. Cell experiments were performed 3 times independently

Differential analysis of the ovarian cancer-related GSE46169 dataset retrieved from the Gene Expression Omnibus (GEO) database revealed three differentially expressed lncRNAs: FIRRE, NEAT1, and ELDR in the tumor samples. Amongst them, the difference multiple of NEAT1 was the largest and NEAT1 also showed up-regulated expression in ovarian cancer (logFC = 4.48, Fig. 1A). Additionally, NEAT1 has been reported to promote the occurrence of ovarian cancer [23], and conversely, let-7 g could inhibit the occurrence of ovarian cancer [16]. Here, RT-qPCR results revealed an enhancement of NEAT1 expression and a decline of let-7 g expression in ovarian cancer tissues in comparison to the adjacent normal tissues (both *p* < 0.05, Fig. 1B, C) (Additional file 1: Table S4). Furthermore, Pearson correlation analysis revealed a negative correlation of NEAT1 expression with let-7 g expression in clinical samples (*p* < 0.05, Fig. 1D). In comparison to the normal ovarian epithelial cell line IOSE80, NEAT1 expression was elevated whereas let-7 g expression was reduced in ovarian cancer cell lines ES2, A2780, HO8910 and SKOV3 (all *p* < 0.05, Fig. 1E, F). The abovementioned results cumulatively indicated that NEAT1 was up-regulated and let-7 g was down-regulated in ovarian cancer, and that they were negatively correlated with each other.

### NEAT1 competitively binds to let-7 g

**Fig. 2:**
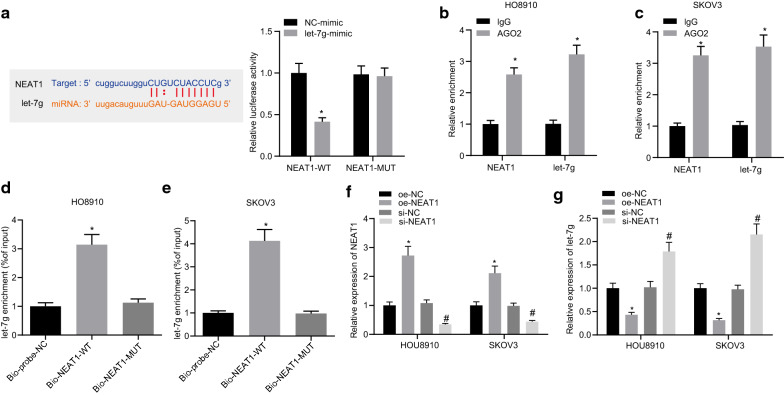
NEAT1 competitively combines to let-7 g. **A** The binding site of let-7 g and NEAT1 predicated using starBase and verified by a dual luciferase reporter gene assay. *p < 0.05 vs. the NC-mimic group. **B** The binding of NEAT1 and let-7 g with AGO2 in HO8910 cells tested by RIP assay. **C** The binding of NEAT1 and let-7 g with AGO2 in SKOV3 cells tested by RIP assay. * *p* < 0.05 vs. the IgG group. **D** The interaction of NEAT1 and let-7 g in HO8910 cells tested by RNA pull down assay. **E** The interaction of NEAT1 and let-7 g in SKOV3 cells tested by RNA pull down assay. *p < 0.05 vs. the Bio-probe NC group. **F** The let-7 g expression in HO8910 cells transfected with si-NEAT1 or oe-NEAT1 tested by RT-qPCR. G, The let-7 g expression in SKOV3 cells transfected with si-NEAT1 or oe-NEAT1 tested by RT-qPCR. *p < 0.05 vs. the oe-NC group, #*p* < 0.05 vs. the si-NC group. The measurement data were expressed as mean ± standard deviation. Comparisons between two groups were conducted by independent sample *t*-test, while comparisons between multiple groups were assessed by one-way ANOVA followed by Tukey's post hoc test. Cell experiments were repeated 3 times

In order to further explore the regulatory mechanism underlying the interaction between NEAT1 and let-7 g, the binding sites between NEAT1 and let-7 g were predicted using starBase. For further validation, a dual luciferase reporter gene assay was performed and the results verified that (Fig. 2A) in comparison to the mimic NC group, the luciferase activity of WT-NEAT1 was decreased in HEK293T cells of the let-7 g mimic group (*p* < 0.05) while that of MUT-NEAT1 showed no significant difference (*p* > 0.05). In addition, RIP assay results demonstrated that (Fig. 2B, C), in HO8910 and SKOV3 cells, Ago2 could enrich more NEAT1 and let-7 g. Furthermore, RNA-pull down assay data established that (Fig. 2D, E) in HO8910 and SKOV3 cells, in comparison to the Bio-probe NC group, the enrichment of let-7 g was markedly increased in the Bio-NEAT1-WT group (*p* < 0.05) while no significant difference was noted in the Bio- NEAT1-MUT group (*p* > 0.05). Moreover, the results of RT-qPCR analysis revealed that (Fig. 2F, G) the expression of let-7 g was enhanced in HO8910 and SKOV3 cells with si-NEAT1 whereas it was found to be reduced in cells with oe-NEAT1. These results demonstrated that overexpression of NEAT1 could potentially suppress the expression of let-7 g.

### NEAT1 augments the growth, migration and invasion of ovarian cancer cells by inhibiting let-7 g expression

**Fig. 3:**
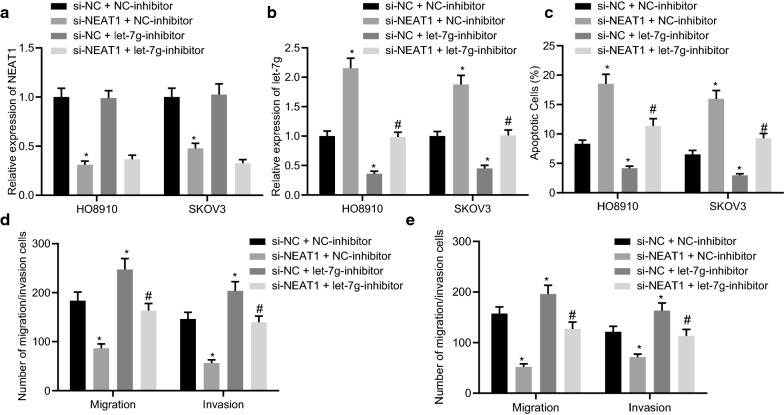
NEAT1 facilitates the growth, migration, and invasion of ovarian cancer cells by inhibiting let-7 g. HO8910 and SKOV3 cells were transfected with si-NEAT1, let-7 g inhibitor or both. **A** Expression of NEAT1 in HO8910 and SKOV3 cells tested by RT-qPCR. **B** Expression of let-7 g in HO8910 and SKOV3 cells quantified by RT-qPCR. **C** The apoptosis of HO8910 and SKOV3 cells measured by flow cytometry. **D** The migration and invasion ability of HO8910 cells assessed by Transwell assay. **E** The migration and invasion ability of SKOV3 cells assessed by Transwell assay. *p < 0.05 vs. the si-NC + NC-inhibitor group. #*p* < 0.05 vs. the si-NEAT1 + NC-inhibitor group. Measurement data were depicted as mean ± standard deviation, and assessed by one-way ANOVA followed by Tukey's post hoc test. Cell experiments were performed 3 times independently

The aforementioned results established that NEAT1 could suppress the expression of let-7 g, and we thus considered that NEAT1 might promote the growth, migration, and invasion of ovarian cancer cells by inhibiting the expression of let-7 g. The results of RT-qPCR displayed that in comparison to the si-NC + NC-inhibitor group, NEAT1 expression was significantly decreased, whereas let-7 g expression was found to be elevated in HO8910 and SKOV3 cells of the si-NEAT1 + NC-inhibitor group (both *p* < 0.05). Versus the si-NC + NC-inhibitor group, the expression of NEAT1 did not show any significant change (*p* > 0.05) and let-7 g expression was found to be reduced in the HO8910 and SKOV3 cells of the si-NC + let-7 g-inhibitor group (*p* < 0.05). Furthermore, in comparison to the si-NEAT1 + NC-inhibitor group, the expression of NEAT1 did not show any significant change (*p* > 0.05) and the let-7 g expression was found to be diminished in the HO8910 and SKOV3 cells of the si-NEAT1 + let-7 g-inhibitor group (*p* < 0.05) (Fig. 3A, B). Moreover, flow cytometric data showed that the silencing of NEAT1 induced the apoptosis of HO8910 and SKOV3 cells, which was inhibited in the presence of let-7 g knockdown or both NEAT1 and let-7 g knockdown (Fig. 3C). Additionally, the results of the Transwell assay revealed that the silencing of NEAT1 suppressed the migration and invasion abilities of HO8910 and SKOV3 cells, which was reversed following let-7 g knockdown or simultaneous knockdown of NEAT1 and let-7 g (Fig. 3D, E). Cumulatively, these data indicated that NEAT1 promoted the growth, migration, and invasion of ovarian cancer cells by inhibiting the expression of let-7 g.

### NEAT1 regulates the MEST/ATGL axis by let-7 g

In order to further investigate the downstream regulatory mechanism of let-7 g, starBase database and mirwalk website were performed to predict the target genes of let-7 g, and GEPIA website was applied to download the list of up-regulated genes in ovarian cancer in TCGA database. After that, the intersection of these predicted genes was obtained, and found that the possible regulatory mRNAs were STON2 and MEST (Additional file 2: Fig. S1). Then, we validated the correlation between the two genes (STON2 and MEST) and let-7 g, revealing that only MEST was negatively correlated with let-7 g (Additional file 3: Fig. S2 and Additional file 4: Fig. S3). Thus, MEST was selected as the gene of interest in our subsequent study.

**Fig. 4:**
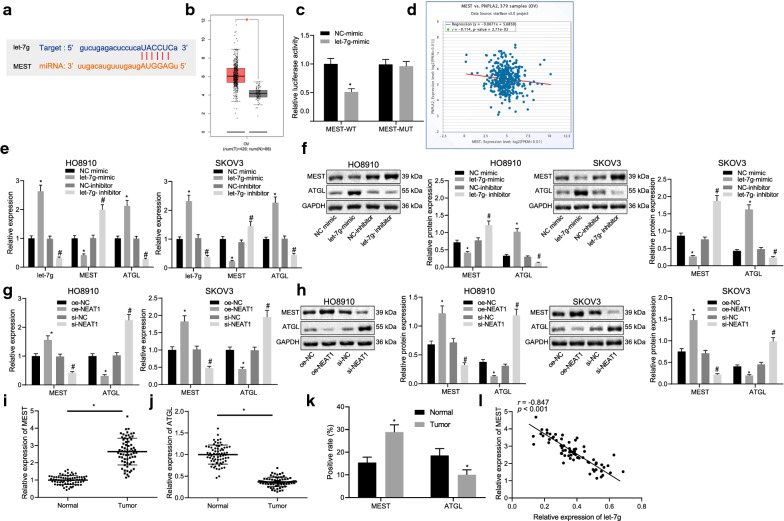
NEAT1 regulates the MEST/ATGL axis by regulating let-7 g. **A** The binding site of let-7 g and MEST predicted using starBase. **B** MEST expression in ovarian cancer samples in the TCGA database. **C** The binding of let-7 g to MEST verified by dual luciferase reporter gene assay. *p < 0.05 vs. the NC-mimic group. **D** Co-expression analysis of MEST and ATGL expression in ovarian cancer samples within the TCGA database. The x-axis represents the expression of MEST, and the y-axis represents the expression of ATGL. **E** The expression of let-7 g, MEST and ATGL in HO8910 and SKOV3 cells after silencing of let-7 g determined by RT-qPCR. **F** The protein expression of MEST and ATGL in HO8910 and SKOV3 cells after silencing of let-7 g determined by western blot analysis. **G** The expression levels of MEST and ATGL in HO8910 and SKOV3 cells after silencing NEAT1, as determined by RT-qPCR. **H** The protein expression of MEST and ATGL in HO8910 and SKOV3 cells after silencing NEAT1 determined by western blot analysis. **I** MEST expression in ovarian cancer tissues and adjacent normal tissues determined by RT-qPCR, n = 68. J, ATGL expression in ovarian cancer tissues and adjacent normal tissues determined by RT-qPCR, n = 68. **K** Positive expression of MEST and ATGL proteins in ovarian cancer tissues and adjacent normal tissues determined by immunohistochemistry. **L** Pearson correlation analysis of let-7 g expression with MEST expression in clinical samples, n = 68. In figure **D**, **E** *p < 0.05 vs. the NC-mimic group. #*p* < 0.05 vs. the NC-inhibitor group. In figure **F**, **G** *p < 0.05 vs. the oe-NC group. #*p* < 0.05 vs. the si-NC group. In figure **H**, **I** *p < 0.05 vs. adjacent normal tissues. The measurement data were expressed as mean ± standard deviation. Comparisons between two groups were conducted by *t*-test, while comparisons among multiple groups were assessed by one-way ANOVA followed by Tukey's post hoc test. All cell experiments were performed 3 times independently

Next, the starBase website was employed to predict the binding sites between let-7 g and MEST (Fig. 4A). Analysis of TCGA database revealed that the expression of MEST was up-regulated in ovarian cancer patients (Fig. 4B), but the effect of MEST on ovarian cancer required further investigation. A dual luciferase reporter gene assay was adopted to verify whether MEST was a direct target of let-7 g. The results demonstrated that, in contrast with the NC mimic group, the luciferase activity of WT-MEST was decreased in the HEK293T cells of the let-7 g mimic group (*p* < 0.05) while that of MUT-MEST showed no significant difference (*p* > 0.05) (Fig. 4C). Furthermore, the silencing of MEST has been previously reported to enhance ATGL expression during the human adipocyte differentiation [19]. Here, the co-expression analysis results suggested that MEST was negatively correlated with ATGL in ovarian cancer samples (Fig. 4D). Previous evidence has shown that ATGL could inhibit the occurrence of ovarian cancer [21]. Therefore, the present study speculated that let-7 g could potentially promote the expression of ATGL by targeting MEST. The results of RT-qPCR and western blot analysis indicated that the up-regulation of let-7 g restrained the expression of MEST but promoted that of ATGL in the HO8910 and SKOV3 cells, which was reversed following depletion of let-7 g (Fig. 4E, F). The abovementioned results suggested that NEAT1 could competitively bind to let-7 g, and in order to further explore whether NEAT1 regulated the MEST/ATGL axis by modulating let-7 g, RT-qPCR and western blot analysis were conducted to determine the expression of MEST and ATGL following silencing of NEAT1 and the results revealed that highly expressed NEAT1 elevated MEST expression while suppressing ATGL expression in the HO8910 and SKOV3 cells. By contrast, poorly expressed NEAT1 promoted ATGL expression but inhibited MEST expression in the HO8910 and SKOV3 cells (Fig. 4G, H). The results of RT-qPCR and immunohistochemistry revealed that in comparison to the adjacent normal tissues, the expression of MEST was heightened while ATGL expression was decreased in the ovarian cancer tissues (both *p* < 0.05) (Fig. 4I–K). Meanwhile, Pearson correlation analysis indicated the inverse correlation of let-7 g with MEST in the clinical samples of ovarian cancer (*p* < 0.05) (Fig. 4L). These results displayed that NEAT1 regulated the MEST/ATGL axis by modulating let-7 g.

### let-7 g attenuates the growth, migration and invasion of ovarian cancer cells by regulating the MEST/ATGL axis

**Fig. 5:**
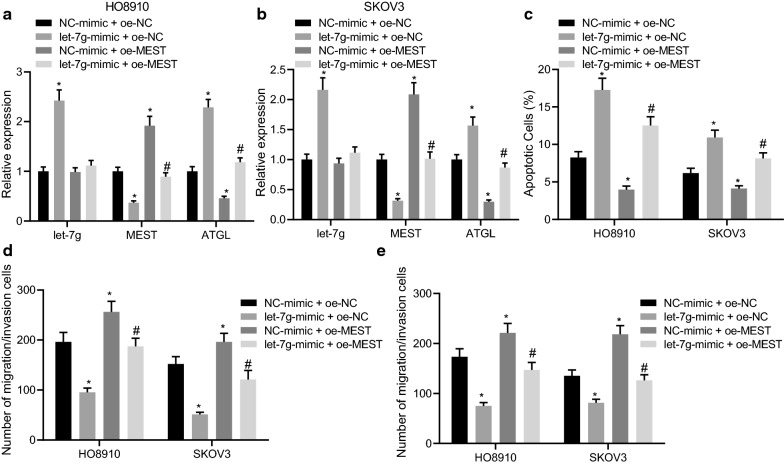
let-7 g attenuates the growth, migration, and invasion of ovarian cancer cells by regulating the MEST/ATGL axis. HO8910 and SKOV3cells were transfected with let-7 g mimic, oe-MEST or both. **A** Expression levels of let-7 g, MEST and ATGL in HO8910 cells determined by RT-qPCR. **B** Expression of let-7 g, MEST, and ATGL in SKOV3 cells determined by RT-qPCR. **C** Apoptosis of HO8910 and SKOV3 cells tested by flow cytometry. **D** Migration and invasion of HO8910 cells determined by Transwell assay. **E** Migration and invasion of SKOV3 cells determined by Transwell assay. *p < 0.05 vs. the NC-mimic + oe-NC group. #*p* < 0.05 vs. the let-7 g-mimic + oe-NC group. Measurement data were depicted as mean ± standard deviation, and assessed by one-way ANOVA followed by Tukey's post hoc test. All cell experiments were performed 3 times independently

The abovementioned results established that NEAT1 promoted the growth, migration, and invasion of ovarian cancer cells by inhibiting let-7 g and regulating the MEST/ATGL axis. The next step was to verify whether let-7 g inhibited the growth, migration, and invasion of ovarian cancer cells by regulating the MEST/ATGL axis. As NEAT1 was found to competitively bind to let-7 g and inhibit its expression, it was speculated that NEAT1 might facilitate the growth, migration, and invasion of ovarian cancer cells by inhibiting let-7 g. The results of RT-qPCR showed that in comparison to the NC-mimic + oe-NC group, let-7 g and ATGL expression was up-regulated but MEST expression was down-regulated in the HO8910 and SKOV3 cells of the let-7 g-mimic + oe-NC group (all *p* < 0.05). Furthermore, in comparison to the si-NC + NC-inhibitor group, let-7 g showed no significant difference (*p* > 0.05), while the expression of MEST was found increased, whereas ATGL expression was decreased in the HO8910 and SKOV3 cells of the NC-mimic + oe-MEST group (both *p* < 0.05). Moreover, in relation to the let-7 g-mimic + oe-NC group, the expression of MEST was enhanced, while the expression of ATGL was decreased in the HO8910 and SKOV3 cells of the let-7 g-mimic + oe-MEST group (both *p* < 0.05) (Fig. 5A, B). Flow cytometric analysis revealed that upon overexpression of let-7 g, the apoptosis of the HO8910 and SKOV3 cells was enhanced, whereas up-regulation of MEST or concomitant up-regulation of MEST and let-7 g reduced cell apoptosis (Fig. 5C). Further, the Transwell assay indicated that overexpression of let-7 g attenuated the migration and invasion abilities of the HO8910 and SKOV3 cells, which were negated following MEST overexpression or concomitant up-regulation of let-7 g and MEST (Fig. 5D, E). These findings demonstrated the inhibitory effect of let-7 g on the growth, migration, and invasion of ovarian cancer cells by regulating the MEST/ATGL axis.

### Silencing of NEAT1 restrains tumorigenesis through regulation of the let-7 g/MEST/ATGL axis in vivo

**Fig. 6:**
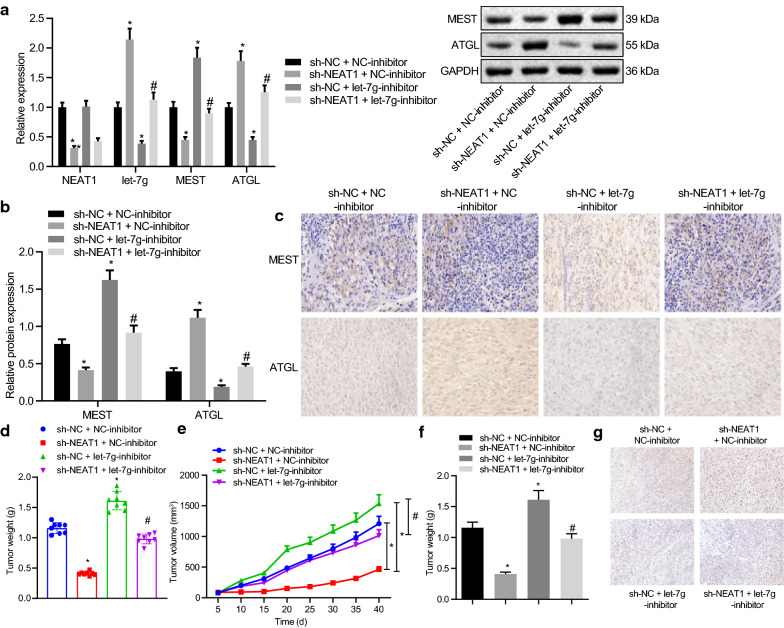
Silencing of NEAT1 restrains in vivo tumorigenesis by regulating the let-7 g/MEST/ATGL axis. Mice were treated with sh-NEAT1, let-7 g inhibitor or both. **A** The expression levels of NEAT1, let-7 g, MEST and ATGL in tumor tissues determined by RT-qPCR. **B** The protein expression of MEST and ATGL in tumor tissues determined by western blot analysis. **C** The positive expression of MEST and ATGL proteins in tumor tissues by immunohistochemistry (400 ×). **D** Representative images of tumors obtained after 40 d of tumor xenograft in nude mice. **E** Tumor growth curve. **F** The final tumor weight in each group. **G** Cell apoptosis of tumor tissues quantified by TUNEL staining (400 ×). *p < 0.05 vs. the si-NC + NC-inhibitor group. #*p* < 0.05 vs. the si-NEAT1 + NC-inhibitor group. Measurement data were depicted as mean ± standard deviation, and assessed by one-way ANOVA followed by Tukey's post hoc test. The data of each group at different time points were compared using repeated measures ANOVA followed by Bonferroni post hoc test

Finally, we aimed to characterize the effect of NEAT1 on the tumorigenesis of ovarian cancer cells via the let-7 g/MEST/ATGL axis in vivo. The results shown in Fig. 6A–C revealed that knockdown of NEAT1 reduced the expression of MEST and elevated the expression levels of let-7 g and ATGL in mouse tumor tissues (Fig. 6AC). Additionally, the silencing of let-7 g raised the expression levels of MEST while decreasing the ATGL expression in mouse tumor tissues. Furthermore, the simultaneous silencing of let-7 g and NEAT1 was noted to reverse the effect of NEAT1 knockdown. Moreover, the knockdown of NEAT1 reduced tumor volume and weight, while silencing of let-7 g led to the opposite results. In addition, simultaneous silencing of let-7 g and NEAT1 abolished the effects of the previous NEAT1 knockdown (Fig. 6D–F). Meanwhile, TUNEL staining data proved that NEAT1 knockdown increased the apoptosis of tumor cells, which was undermined by the subsequent inhibition of let-7 g or simultaneous inhibition of let-7 g and NEAT1 (Fig. 6G). The abovementioned results indicated that silencing of NEAT1 contributed to the promotion of cell apoptosis and inhibition of in vivo tumorigenesis of ovarian cancer cells by regulating the let-7 g/MEST/ATGL axis.

## Discussion

Ovarian cancer is the most fatal gynecologic malignancy owning to typically late diagnosis, lack of definitive curative therapy, and relatively asymptomatic nature in earlier stages [24]. Accumulating evidence has confirmed the involvement of miRNAs in numerous biological processes in cancer cells [25] and also the extensive implication of lncRNAs in tumorigenesis, including in ovarian cancer [26]. The cumulative findings from the current study demonstrated the promoting effects of NEAT1 on the growth, migration, and invasion of ovarian cancer cells whereby NEAT1 competitively bound to let-7 g, and subsequently attenuated its regulatory role in targeting MEST, and down-regulated ATGL expression.

The current study established that the expression of NEAT1 was up-regulated whereas that of let-7 g was decreased in ovarian cancer tissues and cells. Furthermore, NEAT1 could stimulate the growth, migration, and invasion of ovarian cancer cells by inhibiting the expression of let-7 g. Consistent with the present findings, another study has previously reported significantly elevated NEAT1 expression in ovarian cancer tissues and cells [27]. Yet another study also established that the expression of NEAT1 was markedly heightened in ovarian cancer tissues in comparison to the corresponding adjacent non-neoplastic tissues [28]. Furthermore, silencing of NEAT1 has been found to markedly inhibit the invasion of ovarian cancer cells in vitro and attenuate tumor growth in vivo [23]. The down-regulation of NEAT1 has been noted to suppress cell proliferation and facilitate apoptosis of ovarian cancer cells [29]. The expression of let-7 g has been found reduced in other malignancies including human breast cancer samples and hepatocellular carcinoma [14, 15]. let-7 g is also found to be down-regulated in tumor tissue samples of patients with epithelial ovarian cancer in comparison to their non-tumor counterparts, and purportedly it acts as a tumor suppressor to inhibit the development of epithelial ovarian cancer tumor [16]. let-7d-5p has been proven to repress viability, migration, and cell cycle progression of ovarian cancer cells by targeting HMGA1 [30].

LncRNAs can function to reduce the expression of miRNAs by absorbing them through complementary base pairing [31]. The bioinformatic analysis and dual luciferase reporter gene assay employed in the current study confirmed NEAT1 as an inhibitor that down-regulates the expression of let-7 g by competitive binding. Similarly, a recent study reported the binding of miR-let-7b to NEAT1 as well as a negative correlation of NEAT1 expression with miR-let-7b expression in hepatocellular carcinoma tissues [32]. In addition, let-7 g-5p is a downstream target of NEAT1 and can be negatively regulated by NEAT1 in glioblastoma stem cells [12]. Furthermore, NEAT1 has also been found to serve as a competing endogenous RNA against let-7a, which can diminish its expression in the context of non-small cell lung cancer [33]. Therefore, targeting the NEAT1-let-7 g axis might be an effective strategy for the treatment of ovarian cancer.

The present study revealed that let-7 g could target MEST and subsequently inhibit its expression. Partially in line with the current results, let-7 was found to have an inverse correlation with MEST expression during the development of osteosarcoma [34]. Moreover, MEST expression has been demonstrated to be up-regulated in ovarian cancer tissues [35], and is also markedly up-regulated in breast cancer tissues where its inhibition can attenuate breast cancer cell proliferation [36]. In conjunction, a previously conducted study has displayed that the knockdown of MEST reduces cell proliferation whereas promoting the apoptosis of cancer cells [37]. However, the role of MEST in ovarian cancer warrants further exploration. Studies have confirmed that lncRNAs can act as miRNA sponges, and therefore reduce their regulatory effects on the target mRNAs [38, 39], which concurs with the current results showing that NEAT1 up-regulated the expression of the let-7 g target MEST by binding to let-7 g. Additionally, concurrent with the findings of the present study, silencing of MEST was shown to elevate ATGL expression during human adipocyte differentiation [19]. The ATGL gene has been frequently deleted in various cancers, including ovarian, breast and gastric cancers [21]. ATGL and its products (DAG and FFA) have been shown to be responsible for NEAT1-mediated hepatocellular carcinoma cell growth [40]. Additionally, several miRs have demonstrated promotive effects on the expression of ATGL, including miR-27b and miR-34a [41, 42]. However, the effect of let-7 g on ATGL remains unclear and necessitates further investigation.

## Conclusions

**Fig. 7:**
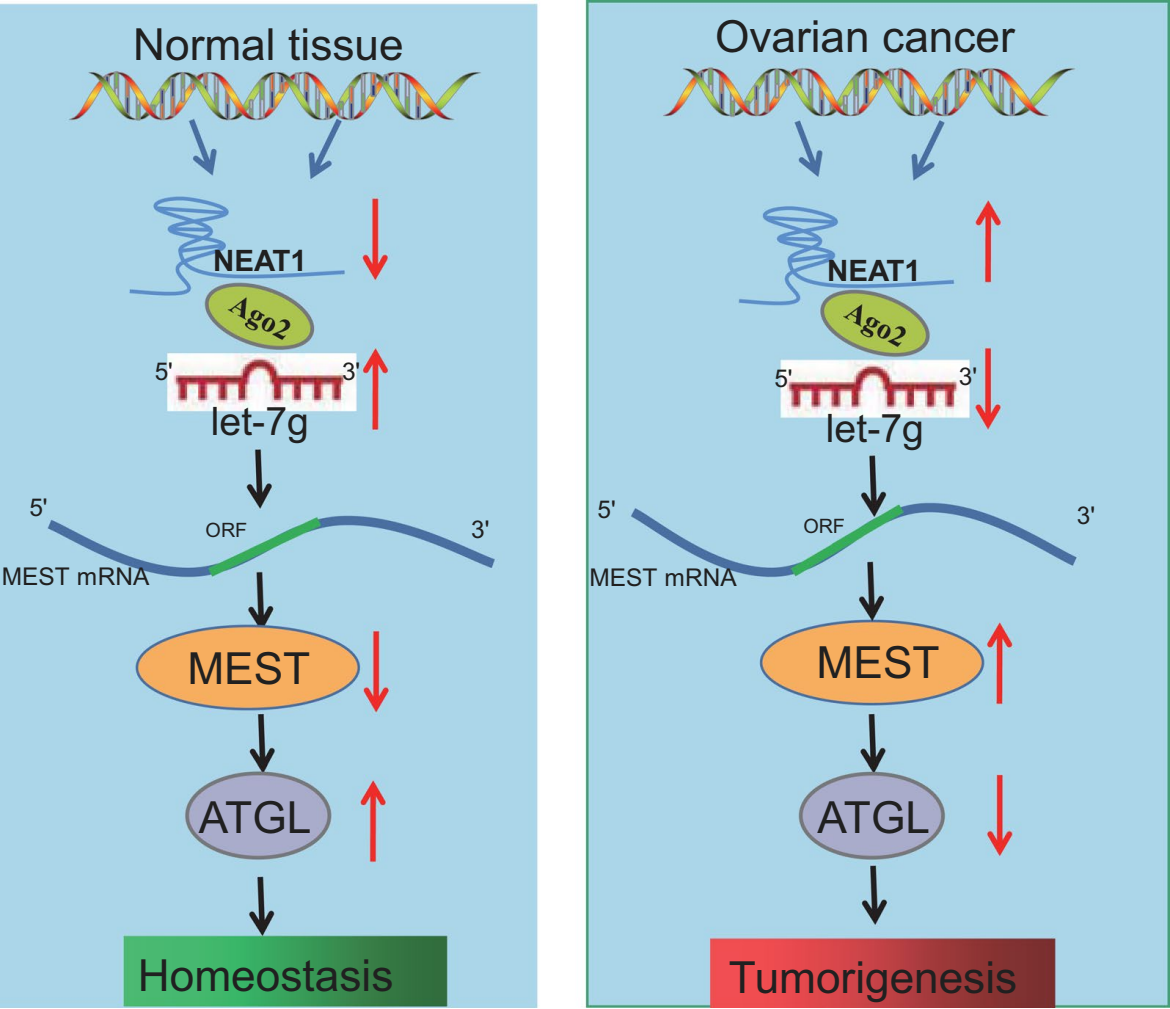
Schematic diagram depicting the mechanism by which NEAT1 is involved in ovarian cancer. NEAT1 competitively binds to let-7 g and inhibits its expression, thereby promoting MEST expression and inhibiting ATGL expression, and ultimately promoting the growth, migration, and invasion of ovarian cancer cells as well as tumor metastasis

In conclusion, the current study provides evidence indicating that NEAT1 can potentially promote the growth, migration, and invasion of ovarian cancer cells in vitro as well as tumor growth in vivo by competitively binding to let-7 g, promoting MEST expression and inhibiting ATGL expression (Fig. 7). These findings suggest that targeting NEAT1 may enable the development of novel therapeutics for the prevention and treatment of ovarian cancer. However, whether other miRNAs known to interact with NEAT1 in ovarian cancer can also target the MEST or ATGL genes requires further comprehensive investigations.

## Supplementary Information

**Additional file 1: Table S1.** Correlation of NEAT1, let-7 g and MESTD expression with the clinical characteristics of ovarian cancer patients. **Table S2.** STR profiling of ovarian cancer cell lines. **Table S3.** Primer sequences used for RT-qPCR. **Table S4.** Original data of expression of NEAT1, let-7g, MEST, and ATGL in ovarian cancer tissues and adjacent normal tissues of patients.

**Additional file 2: Fig. S1.** The intersection of target genes of let-7g predicted in starBase and mirwalk databases and up-regulated genes in ovarian cancer in TCGA database.

**Additional file 3: Fig. S2.** The correlation of MEST with let-7g in ovarian cancer analyzed by starBase database.

**Additional file 4: Fig. S3.** The correlation of STON2 with let-7g in ovarian cancer analyzed by starBase database.

## Data Availability

The datasets supporting the conclusions of this article are available.

## Abbreviations

lncRNAs: Long non-coding RNAs
NEAT1: Nuclear paraspeckle assembly transcript 1
MEST: Mesoderm specific transcript
ATGL: Adipose triglyceride lipase
STR: Short tandem repeat
DMEM: Dulbecco’s modified Eagle’s medium
NCBI: National Center for Biotechnology Information
cDNA: Complementary DNA
IgG: Immunoglobulin G
WT: Wild type
MUT: Mutant type
AGO2: Argonaute2
FBS: Fetal bovine serum
si-NEAT: Small interfering RNA targeting NEAT
RT-qPCR: Reverse transcription quantitative polymerase chain reaction
PBS: Phosphate-buffered saline
GAPDH: Glyceraldehyde-3-phosphate dehydrogenase
RIPA: Radio-immunoprecipitation assay
POD: Peroxidase
DAB: 3,3'-Diaminobenzidine tetrahydrochloride
UTR: Untranslated region
RIP: RNA binding protein immunoprecipitation
oe: Overexpression
NC: Negative control
sh: Short hairpin
TUNEL: Terminal deoxynucleotidyl transferase-mediated deoxyuridine triphosphate-biotin nick end-labeling
GEO: Gene Expression Omnibus
ANOVA: Analysis of variance
