# Supplementary material for: Long non-coding RNA NEAT1 facilitates the growth, migration, and invasion of ovarian cancer cells via the let-7 g/MEST/ATGL axis
Source: Cancer Cell Int. 2021 Aug 20;21:437. doi: 10.1186/s12935-021-02018-3 (PMC8379830; doi:10.1186/s12935-021-02018-3)
Supplement: Supplementary file 1 — Additional file 1: Table S1. Correlation of NEAT1, let-7 g and MESTD expression with the clinical characteristics of ovarian cancer patients. Table S2. STR profiling of ovarian cancer cell lines. Table S3. Primer sequences used for RT-qPCR. Table S4. Original data of expression of NEAT1, let-7g, MEST, and ATGL in ovarian cancer tissues and adjacent normal tissues of patients. [file 12935_2021_2018_MOESM1_ESM.docx]

**Supplementary Table 1.** Correlation of NEAT1, let-7g and MESTD expression with the clinical characteristics of ovarian cancer patients.

| Items | n | NEAT1 expression | | *p* | let-7g expression | | *p* | MESTD expression | | *p* |
| --- | --- | --- | --- | --- | --- | --- | --- | --- | --- | --- |
|  |  | Low | High |  | Low | High |  | Low | High |  |
| Age |  |  |  | 0.451 |  |  | 0.451 |  |  | 0.801 |
| < 50 |  | 20 | 23 |  | 20 | 23 |  | 21 | 22 |  |
| ≥ 50 |  | 14 | 11 |  | 14 | 11 |  | 13 | 12 |  |
| FIGO stage |  |  |  | 0.002 |  |  | < 0.001 |  |  | 0.029 |
| I-II |  | 24 | 11 |  | 10 | 25 |  | 22 | 13 |  |
| III |  | 10 | 23 |  | 24 | 9 |  | 12 | 21 |  |
| Lymph node metastasis |  |  |  | 0.001 |  |  | < 0.001 |  |  | < 0.001 |
| Yes |  | 5 | 18 |  | 20 | 3 |  | 4 | 19 |  |
| No |  | 29 | 16 |  | 14 | 31 |  | 30 | 15 |  |

**Supplementary Table 2.** STR profiling of ovarian cancer cell lines.

|  | AMEL | D5S818 | D13S317 | D7S820 | D16S539 | vWA | THO1 | TPOX | CSF1PO |
| --- | --- | --- | --- | --- | --- | --- | --- | --- | --- |
| HELA | X | 11,12 | 12,13.3 | 8,12 | 9,10 | 16,18 | 7 | 8,12 | 9,10 |
| A2780 | X | 11,12 | 12,13 | 10 | 11,13 | 15,16 | 6 | 8,10 | 10,11 |
| SKOV3 | X | 11 | 8,11 | 13,14 | 12 | 17,18 | 9,9.3 | 8,11 | 11 |
| ES2 | X | 11,13 | 11 | 11 | 11,13 | 16,17 | 9.3 | 8,12 | 10,15 |
| OVCAR3 | X | 11,12 | 12 | 10 | 12 | 17 | 9.3 | 8 | 11,12 |

**Supplementary Table 3.** Primer sequences used for RT-qPCR.

| Gene | Forward sequence (5’-3’) | Reverse sequence (5’-3’) |
| --- | --- | --- |
| NEAT1 | GACCTCTCACCTACCCACCT | CTTGTACCCTCCCAGCGTTT |
| let-7g | UGAGGTAGTAGTTTGT |  |
| MEST | GTCCTGTAGGCAAGGTCTTACC | AGGTACGCAGCAAGCAGG |
| ATGL | GCTTCCTCGGCGTCTACTAC | CAATGAACTTGGCACCAGCC |
| U6 | GGAACGATACAGAGAAGATTAGC | TGGAACGCTTCACGAATTTGCG |
| GAPDH | CTCTGCTCCTCCTGTTCGAC | GCGCCCAATACGACCAAATC |

Note: NEAT1, nuclear-enriched abundant transcript 1; MEST, mesoderm specific transcript; ATGL, adipose triglyceride lipase; GAPDH, glyceraldehyde-3-phosphate dehydrogenase.

**Table S4** Original data of expression of NEAT1, let-7g, MEST, and ATGL in ovarian cancer tissues and adjacent normal tissues of patients

| Gender | Age | FIGO staging | Lymph node metastasis | Normal | Tumor | Normal | Tumor | Normal | Tumor | Normal | Tumor | Normal | Tumor | Normal | Tumor |
| --- | --- | --- | --- | --- | --- | --- | --- | --- | --- | --- | --- | --- | --- | --- | --- |
| Female | 60 | advanced | yes | 1.465 | 3.566 | 0.899 | 0.184 | 0.495 | 3.963 | 1.292 | 0.167 | 15.180 | 29.980 | 15.070 | 11.650 |
| Female | 22 | early | no | 0.696 | 2.072 | 0.984 | 0.468 | 0.888 | 4.683 | 0.772 | 0.261 | 13.600 | 26.570 | 10.130 | 10.530 |
| Female | 55 | advanced | no | 1.131 | 2.555 | 0.850 | 0.325 | 0.910 | 2.458 | 1.271 | 0.405 | 16.340 | 34.170 | 22.780 | 10.140 |
| Female | 38 | early | no | 1.068 | 0.954 | 1.051 | 0.607 | 1.517 | 1.640 | 1.029 | 0.383 | 15.510 | 30.530 | 19.690 | 10.770 |
| Female | 45 | advanced | yes | 0.728 | 2.326 | 0.644 | 0.317 | 0.882 | 2.745 | 1.075 | 0.390 | 18.640 | 32.310 | 18.800 | 10.260 |
| Female | 45 | early | no | 1.237 | 2.838 | 0.677 | 0.518 | 1.172 | 2.094 | 0.809 | 0.369 | 14.250 | 31.010 | 20.480 | 9.770 |
| Female | 21 | advanced | yes | 1.012 | 3.079 | 0.681 | 0.273 | 0.930 | 3.336 | 0.890 | 0.501 | 20.580 | 25.160 | 24.280 | 9.170 |
| Female | 55 | advanced | no | 0.775 | 2.713 | 0.928 | 0.500 | 0.870 | 2.338 | 1.159 | 0.319 | 11.020 | 29.170 | 17.970 | 8.300 |
| Female | 43 | early | no | 1.215 | 1.516 | 0.814 | 0.520 | 1.218 | 1.455 | 1.144 | 0.336 | 12.360 | 32.190 | 18.760 | 13.360 |
| Female | 55 | early | no | 1.114 | 1.836 | 0.922 | 0.567 | 0.934 | 2.038 | 1.183 | 0.431 | 13.640 | 26.890 | 17.020 | 14.080 |
| Female | 43 | early | no | 0.915 | 0.645 | 1.206 | 0.358 | 0.726 | 1.774 | 1.013 | 0.351 | 20.270 | 26.660 | 13.850 | 9.700 |
| Female | 53 | early | no | 0.525 | 1.348 | 0.788 | 0.386 | 1.249 | 1.982 | 1.278 | 0.316 | 14.530 | 31.350 | 20.170 | 8.710 |
| Female | 41 | advanced | yes | 0.950 | 2.271 | 1.082 | 0.364 | 1.029 | 2.697 | 0.987 | 0.248 | 14.810 | 30.310 | 19.120 | 10.180 |
| Female | 53 | advanced | yes | 0.506 | 3.398 | 0.635 | 0.268 | 1.044 | 3.763 | 1.242 | 0.425 | 13.480 | 25.400 | 23.690 | 13.810 |
| Female | 53 | advanced | yes | 0.958 | 1.769 | 1.032 | 0.305 | 0.878 | 3.530 | 0.824 | 0.580 | 12.070 | 23.890 | 21.010 | 9.980 |
| Female | 38 | early | yes | 1.132 | 2.079 | 1.162 | 0.383 | 1.338 | 2.812 | 1.007 | 0.458 | 18.200 | 30.710 | 17.810 | 9.560 |
| Female | 59 | early | no | 0.763 | 2.055 | 0.830 | 0.372 | 1.279 | 2.563 | 0.684 | 0.407 | 15.490 | 27.870 | 17.430 | 6.810 |
| Female | 56 | advanced | yes | 1.160 | 2.363 | 1.379 | 0.381 | 0.606 | 2.664 | 0.961 | 0.223 | 13.100 | 26.740 | 22.990 | 11.910 |
| Female | 53 | early | no | 1.007 | 3.720 | 0.944 | 0.553 | 0.957 | 3.944 | 1.338 | 0.357 | 15.720 | 29.240 | 15.980 | 8.120 |
| Female | 30 | advanced | yes | 1.090 | 2.538 | 0.859 | 0.147 | 0.777 | 1.869 | 0.903 | 0.328 | 17.300 | 28.870 | 19.960 | 11.390 |
| Female | 64 | advanced | yes | 1.221 | 3.013 | 0.768 | 0.319 | 1.080 | 3.029 | 0.804 | 0.474 | 15.920 | 27.650 | 17.480 | 8.400 |
| Female | 32 | advanced | no | 0.544 | 1.596 | 1.132 | 0.592 | 1.089 | 2.686 | 0.719 | 0.437 | 13.470 | 30.570 | 18.890 | 8.800 |
| Female | 45 | early | yes | 1.057 | 2.529 | 1.040 | 0.518 | 1.439 | 2.025 | 0.950 | 0.253 | 17.390 | 29.100 | 18.990 | 11.840 |
| Female | 37 | early | no | 0.976 | 2.452 | 1.031 | 0.447 | 1.156 | 2.482 | 0.669 | 0.387 | 12.310 | 32.310 | 19.010 | 5.850 |
| Female | 28 | early | no | 0.912 | 1.002 | 0.996 | 0.628 | 1.084 | 2.488 | 1.136 | 0.205 | 15.810 | 34.020 | 19.240 | 9.680 |
| Female | 61 | advanced | no | 1.184 | 4.277 | 1.290 | 0.133 | 1.111 | 2.609 | 0.852 | 0.431 | 13.400 | 24.500 | 23.230 | 10.250 |
| Female | 41 | advanced | yes | 1.320 | 3.513 | 1.141 | 0.131 | 1.174 | 3.347 | 0.880 | 0.377 | 13.050 | 35.760 | 17.670 | 8.680 |
| Female | 48 | early | no | 0.997 | 3.795 | 1.067 | 0.391 | 0.673 | 3.418 | 1.354 | 0.313 | 11.210 | 30.250 | 23.060 | 7.120 |
| Female | 25 | early | no | 0.874 | 2.291 | 0.616 | 0.444 | 1.304 | 2.971 | 0.922 | 0.347 | 16.970 | 30.930 | 16.300 | 8.150 |
| Female | 53 | advanced | yes | 1.427 | 2.689 | 0.801 | 0.355 | 0.715 | 3.571 | 0.937 | 0.354 | 16.680 | 28.420 | 19.590 | 10.870 |
| Female | 57 | early | no | 1.054 | 2.179 | 0.933 | 0.401 | 0.801 | 3.478 | 0.782 | 0.359 | 12.270 | 23.380 | 20.810 | 9.500 |
| Female | 42 | early | no | 1.104 | 2.236 | 1.230 | 0.381 | 0.601 | 3.107 | 1.458 | 0.344 | 16.760 | 30.020 | 19.910 | 9.250 |
| Female | 48 | advanced | no | 0.922 | 1.557 | 0.689 | 0.503 | 1.182 | 2.905 | 1.161 | 0.343 | 17.300 | 28.250 | 13.020 | 6.430 |
| Female | 39 | early | no | 0.942 | 1.689 | 1.177 | 0.550 | 1.047 | 2.310 | 0.903 | 0.520 | 14.870 | 26.450 | 21.460 | 12.160 |
| Female | 62 | advanced | no | 0.587 | 1.625 | 1.385 | 0.560 | 1.597 | 1.313 | 0.927 | 0.317 | 14.730 | 30.350 | 19.700 | 12.190 |
| Female | 57 | early | no | 0.696 | 1.712 | 1.153 | 0.507 | 0.536 | 1.543 | 1.044 | 0.350 | 12.470 | 24.290 | 14.590 | 10.720 |
| Female | 55 | advanced | yes | 0.703 | 2.821 | 1.064 | 0.216 | 1.209 | 3.478 | 0.945 | 0.350 | 16.540 | 30.420 | 20.390 | 11.160 |
| Female | 45 | early | no | 1.153 | 2.391 | 0.972 | 0.437 | 0.874 | 2.511 | 1.197 | 0.392 | 16.630 | 30.900 | 16.020 | 14.360 |
| Female | 29 | advanced | yes | 0.867 | 2.797 | 1.547 | 0.304 | 0.918 | 2.763 | 1.228 | 0.535 | 11.380 | 29.510 | 12.370 | 8.010 |
| Female | 39 | early | no | 0.850 | 2.044 | 0.841 | 0.414 | 0.431 | 3.686 | 0.972 | 0.225 | 16.780 | 34.440 | 20.410 | 10.560 |
| Female | 38 | advanced | yes | 0.852 | 2.804 | 1.074 | 0.308 | 0.774 | 2.215 | 1.208 | 0.346 | 14.350 | 28.930 | 18.040 | 8.920 |
| Female | 45 | early | no | 0.924 | 1.487 | 0.568 | 0.685 | 0.629 | 1.524 | 0.728 | 0.435 | 18.580 | 28.910 | 16.300 | 7.620 |
| Female | 41 | advanced | no | 0.763 | 2.799 | 0.869 | 0.480 | 1.127 | 1.386 | 0.497 | 0.366 | 17.960 | 30.300 | 17.580 | 8.760 |
| Female | 41 | early | no | 0.804 | 3.273 | 0.956 | 0.359 | 1.366 | 2.291 | 1.168 | 0.480 | 13.520 | 27.840 | 14.150 | 12.680 |
| Female | 41 | early | no | 1.120 | 3.470 | 0.735 | 0.329 | 0.571 | 2.584 | 1.106 | 0.442 | 16.720 | 26.580 | 17.160 | 9.350 |
| Female | 37 | advanced | no | 1.017 | 2.666 | 1.010 | 0.250 | 1.176 | 1.216 | 0.699 | 0.559 | 18.050 | 25.440 | 19.900 | 6.120 |
| Female | 27 | advanced | yes | 1.132 | 3.299 | 0.980 | 0.230 | 0.747 | 3.389 | 1.178 | 0.344 | 14.150 | 29.360 | 20.970 | 12.710 |
| Female | 47 | early | no | 1.260 | 2.543 | 1.256 | 0.191 | 1.106 | 3.598 | 0.463 | 0.315 | 12.970 | 23.570 | 20.580 | 9.160 |
| Female | 42 | early | no | 0.965 | 2.386 | 1.271 | 0.177 | 1.018 | 3.715 | 1.097 | 0.384 | 18.490 | 33.650 | 15.780 | 11.690 |
| Female | 41 | advanced | no | 0.867 | 1.511 | 0.955 | 0.379 | 1.028 | 2.208 | 1.341 | 0.309 | 14.170 | 30.590 | 18.020 | 8.550 |
| Female | 32 | advanced | yes | 1.066 | 2.461 | 0.865 | 0.349 | 1.067 | 2.656 | 1.027 | 0.492 | 15.550 | 21.330 | 18.980 | 10.830 |
| Female | 64 | early | no | 0.949 | 2.378 | 1.132 | 0.392 | 0.871 | 2.447 | 0.886 | 0.297 | 10.340 | 34.910 | 18.500 | 9.320 |
| Female | 32 | early | no | 0.917 | 2.939 | 1.316 | 0.352 | 0.955 | 2.567 | 1.071 | 0.332 | 17.980 | 29.810 | 18.550 | 9.810 |
| Female | 41 | advanced | yes | 1.258 | 2.539 | 1.339 | 0.306 | 0.923 | 3.225 | 1.603 | 0.549 | 20.820 | 29.180 | 15.230 | 8.250 |
| Female | 56 | advanced | yes | 1.581 | 3.030 | 1.256 | 0.234 | 1.324 | 3.706 | 0.990 | 0.226 | 16.320 | 27.950 | 20.040 | 12.490 |
| Female | 26 | early | no | 1.063 | 2.179 | 1.244 | 0.314 | 1.146 | 2.739 | 1.022 | 0.283 | 13.140 | 25.850 | 13.220 | 7.650 |
| Female | 57 | early | no | 1.117 | 2.322 | 1.128 | 0.474 | 0.992 | 2.054 | 1.104 | 0.141 | 16.440 | 29.240 | 19.970 | 15.170 |
| Female | 49 | early | no | 0.936 | 2.048 | 0.928 | 0.404 | 0.729 | 2.335 | 0.697 | 0.367 | 14.660 | 23.660 | 13.170 | 11.200 |
| Female | 57 | early | no | 1.078 | 1.602 | 0.918 | 0.523 | 0.716 | 1.144 | 0.925 | 0.688 | 15.320 | 32.500 | 17.380 | 11.750 |
| Female | 30 | early | no | 0.720 | 2.421 | 0.780 | 0.393 | 0.821 | 2.840 | 0.854 | 0.343 | 19.540 | 23.010 | 21.120 | 8.170 |
| Female | 63 | early | no | 1.337 | 1.365 | 1.334 | 0.255 | 1.409 | 3.646 | 0.744 | 0.178 | 16.020 | 28.800 | 21.050 | 12.040 |
| Female | 24 | advanced | no | 1.096 | 2.626 | 0.809 | 0.405 | 0.996 | 2.355 | 0.851 | 0.325 | 15.610 | 23.150 | 16.860 | 9.250 |
| Female | 53 | advanced | yes | 1.110 | 4.833 | 1.114 | 0.226 | 1.385 | 3.203 | 1.033 | 0.163 | 18.950 | 31.540 | 23.360 | 16.650 |
| Female | 57 | advanced | no | 1.149 | 1.869 | 1.113 | 0.215 | 1.174 | 1.923 | 0.661 | 0.473 | 15.140 | 33.910 | 17.760 | 10.060 |
| Female | 30 | advanced | yes | 1.005 | 3.420 | 1.062 | 0.311 | 1.164 | 1.650 | 1.190 | 0.420 | 15.270 | 27.040 | 20.730 | 9.290 |
| Female | 48 | advanced | yes | 1.123 | 2.623 | 0.864 | 0.301 | 1.117 | 3.031 | 1.041 | 0.384 | 12.140 | 31.440 | 17.720 | 6.350 |
| Female | 41 | advanced | no | 1.130 | 1.072 | 1.021 | 0.551 | 1.225 | 2.865 | 0.977 | 0.170 | 17.450 | 25.800 | 25.190 | 8.180 |
| Female | 53 | early | no | 0.827 | 2.200 | 0.860 | 0.354 | 0.719 | 1.550 | 1.135 | 0.574 | 16.370 | 28.340 | 14.600 | 9.580 |
